# Self-energy dynamics and the mode-specific phonon threshold effect in Kekulé-ordered graphene

**DOI:** 10.1093/nsr/nwab175

**Published:** 2021-09-16

**Authors:** Hongyun Zhang, Changhua Bao, Michael Schüler, Shaohua Zhou, Qian Li, Laipeng Luo, Wei Yao, Zhong Wang, Thomas P Devereaux, Shuyun Zhou

**Affiliations:** State Key Laboratory of Low-Dimensional Quantum Physics and Department of Physics, Tsinghua University, Beijing 100084, China; State Key Laboratory of Low-Dimensional Quantum Physics and Department of Physics, Tsinghua University, Beijing 100084, China; Stanford Institute for Materials and Energy Sciences (SIMES), SLAC National Accelerator Laboratory, Menlo Park, CA 94025, USA; State Key Laboratory of Low-Dimensional Quantum Physics and Department of Physics, Tsinghua University, Beijing 100084, China; State Key Laboratory of Low-Dimensional Quantum Physics and Department of Physics, Tsinghua University, Beijing 100084, China; State Key Laboratory of Low-Dimensional Quantum Physics and Department of Physics, Tsinghua University, Beijing 100084, China; State Key Laboratory of Low-Dimensional Quantum Physics and Department of Physics, Tsinghua University, Beijing 100084, China; Institute for Advanced Study, Tsinghua University, Beijing 100084, China; Stanford Institute for Materials and Energy Sciences (SIMES), SLAC National Accelerator Laboratory, Menlo Park, CA 94025, USA; Department of Materials Science and Engineering, Stanford University, Stanford, CA 94035, USA; State Key Laboratory of Low-Dimensional Quantum Physics and Department of Physics, Tsinghua University, Beijing 100084, China; Frontier Science Center for Quantum Information, Beijing 100084, China

**Keywords:** TrARPES, self-energy, electron-phonon coupling, Kekulé-ordered graphene

## Abstract

Electron-phonon interaction and related self-energy are fundamental to both the equilibrium properties and non-equilibrium relaxation dynamics of solids. Although electron-phonon interaction has been suggested by various time-resolved measurements to be important for the relaxation dynamics of graphene, the lack of energy- and momentum-resolved self-energy dynamics prohibits direct identification of the role of specific phonon modes in the relaxation dynamics. Here, by performing time- and angle-resolved photoemission spectroscopy measurements on Kekulé-ordered graphene with folded Dirac cones at the Γ point, we have succeeded in resolving the self-energy effect induced by the coupling of electrons to two phonons at Ω_1_ = 177 meV and Ω_2_ = 54 meV, and revealing its dynamical change in the time domain. Moreover, these strongly coupled phonons define energy thresholds, which separate the hierarchical relaxation dynamics from ultrafast, fast to slow, thereby providing direct experimental evidence for the dominant role of mode-specific phonons in the relaxation dynamics.

## Teaser

High resolution TrARPES measurements of Kekule-ordered graphene resolve the dynamical evolution of electron-phonon interaction in the time domain and reveal the role of specific phonon modes in the ultrafast relaxation dynamics.

## INTRODUCTION

Electron-phonon interaction is ubiquitous in solids and fundamental to the transport properties [1,2] as well as the non-equilibrium relaxation dynamics. Electron-phonon interaction determines the electrical resistivity of metals [1,2], affects the electron mobility of semiconductors and drives phase transitions, such as charge density wave [3] and superconductivity [4,5]. Electron-phonon interaction also plays a critical role in the non-equilibrium relaxation dynamics, as has been revealed by various time-resolved optical measurements, where the relaxation rate of electrons is determined by the electron-phonon coupling strength averaged over all phonons [6–9]. In order to further identify whether the relaxation dynamics is dominantly determined by specific phonon modes that are strongly coupled with electrons or contributed by all phonons, it is important to experimentally resolve the self-energy Σ in the time domain and reveal its dynamic evolution. Angle-resolved photoemission spectroscopy (ARPES) is a powerful tool for extracting the real and imaginary parts of the self-energy ReΣ and |ImΣ|, which show up in the ARPES data as a renormalization of the electronic dispersion [2] and an increase in the scattering rate near the phonon energy. By combining ARPES with ultrafast pump-probe, time-resolved ARPES (TrARPES) provides unique opportunities for revealing the self-energy effect in the time domain with mode-specific information and establishing a direct connection between the strongly coupled phonons and the relaxation dynamics.

Graphene with low-energy excitations resembling relativistic Dirac fermions [10,11] and strong electron-phonon coupling indicated by the Kohn anomaly [12] is a model system for investigating the electron-phonon interaction in both the equilibrium and non-equilibrium states. The electron-phonon coupling-induced self-energy effect has been resolved in the ARPES measurements of graphene and graphite [13–19] and suggested to be important for the carrier relaxation from time-resolved optical measurements [20–22]. While TrARPES measurements have been performed to reveal the relaxation dynamics of photo-excited carriers [23–35], so far, the electron-phonon coupling-induced self-energy effect and the associated self-energy dynamics have not been resolved in any TrARPES measurement of graphene or graphite. Experimentally, this is limited by the reduced efficiency and resolution of the high harmonic generation (HHG) light source [35–37], which is required for generating a sufficiently high photon energy to probe the Dirac cone at the K point with a large momentum value of 1.7 Å^−1^.

**Figure 1.**
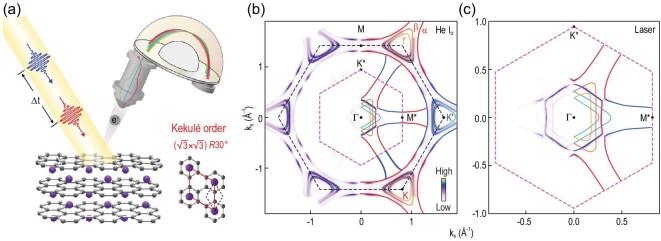
A schematic of TrARPES and a Fermi surface map of Kekulé-ordered graphene. (a) A schematic of TrARPES on Li-intercalated trilayer graphene with a superlattice period of ($\sqrt{3}\times \sqrt{3}$)*R*30^°^. The unit cells for graphene and the Kekulé order are labeled with black and red parallelograms. (b) Fermi surface map of the Li-intercalated trilayer graphene measured with a helium lamp source at 21.2 eV. Red and blue curves indicate the largest pockets (α) at K and K^′^. Smaller pockets β/γ at K and K^′^ are indicated by yellow and green curves. Black and pink dashed hexagons are graphene and superlattice BZs, respectively. Colored curves around Γ indicate folded pockets from the K and K^′^ points. (c) Enlarged Fermi surface map using a 6.2 eV laser source. The largest pockets from the K and K^′^ points are highlighted in red and blue. The pink dashed hexagon is the superlattice BZ with high symmetry points K^*^ and M^*^ labeled.

Here, by taking an experimental strategy of folding the Dirac cones from K to Γ by inducing a $(\sqrt{3}\times \sqrt{3})$*R*30^°^ (Fig. 1a) Kekulé order [38,39] through Li intercalation [40–43], we are able to probe the dynamics of Dirac cones using the photon energy of 6.2 eV with greatly improved momentum resolution. This leads to successful identification of coupling of electrons to phonons at Ω_1_ = 177 meV and Ω_2_ = 54 meV in both ReΣ and |ImΣ| in the time domain. Moreover, these two strongly coupled phonons dominate the relaxation dynamics of electrons by setting energy thresholds for the hierarchical relaxation dynamics from ultrafast, fast to slow. Our work reveals the dynamical modification of the electron-phonon coupling-induced self-energy effect in the time domain and highlights the dominant role of mode-specific electron-phonon interaction in the non-equilibrium dynamics.

## RESULTS

The Kekulé-ordered trilayer graphene sample is obtained by intercalating Li into a graphene sample grown on a SiC substrate [40–43], as schematically shown in Fig. S1 of the online supplementary material. The intercalation leads to AA stacking [42] with characteristic dispersion shown in Fig. S2 of the online supplementary material. The $(\sqrt{3}\times \sqrt{3})R30^\circ$ Kekulé order not only leads to a replica of Dirac cones at the Γ point, but also leads to the intervalley coupling and a chiral symmetry breaking induced gap opening [43], which is in analogy to the dynamical mass generation in particle physics. In this work, we focus on the electronic dynamics of the folded Dirac cones at the Γ point of a Li-intercalated trilayer graphene by TrARPES measurements using a 6.2 eV probe laser source operating at a higher repetition rate, which leads to a much higher experimental efficiency and at least a 3 times improvement in the momentum resolution compared to previous TrARPES measurements with a HHG light source (for more information about momentum and time resolution, see the Methods section and Fig. S3 of the online supplementary material). Such improvement is critical for successfully resolving the self-energy effect in the TrARPES measurements.

Figure 1b shows the Fermi surface map measured by a helium lamp source, which contains three large Fermi pockets (indicated by α, β and γ, and colored curves) with different sizes around each Brillouin zone (BZ) corner (for more information about the Fermi surface map and dispersion image, see Fig. S2 of the online supplementary material). The large pocket size indicates large electron doping induced by the intercalated Li. Folded Dirac cones by the $(\sqrt{3}\times \sqrt{3})R30^\circ$ Kekulé superlattice are observed at the Γ point, similar to previous work [43], and these folded pockets are better resolved in the enlarged Fermi surface map measured by using a laser source with the photon energy of 6.2 eV (Fig. 1c). We note that the Li-intercalated monolayer graphene does not show folded Dirac cones at the Γ point [44], and the Li-intercalated trilayer graphene sample shows a much stronger intensity for the folded pockets at the Γ point than the Li-intercalated bilayer graphene sample [43]. Therefore, a Li-intercalated trilayer graphene sample is used in this TrARPES study. Considering that the Li-intercalated samples are arranged in the AA stacking sequence with weak interlayer coupling, the physics of Li-intercalated trilayer and bilayer graphene is expected to be similar.

**Figure 2.**
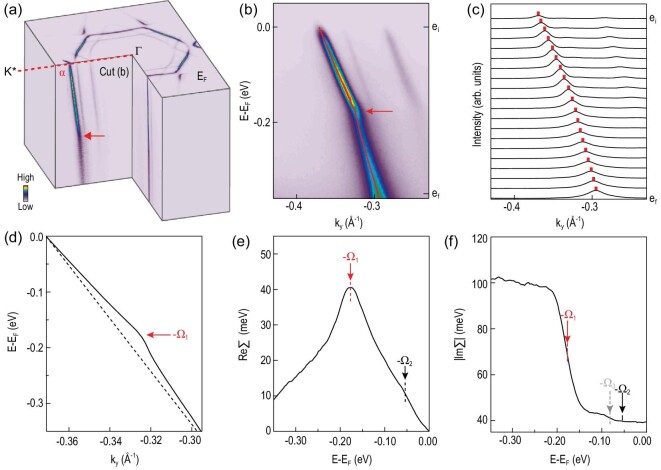
Electron-phonon coupling-induced self-energy effect. (a) Three-dimensional band structure of the Kekulé-ordered graphene measured using a 6.2 eV laser source. The red arrow indicates the kink, and the red dashed line indicates the Γ–K^*^ (Γ–M) direction. (b) Dispersion image measured along the direction indicated by the red dashed line in (a) before pump excitation. The dispersing band with the strongest intensity is from the largest Fermi surface pocket α. (c) MDCs at energies labeled by e_*i*_ to e_*f*_ in (b); the red marks indicate the peak positions from Lorentzian fitting. (d) Extracted dispersion from fitting the MDCs in (c). Black dotted line indicates the bare band dispersion used for extracting ReΣ in (e). (e) and (f) Extracted ReΣ and |ImΣ| reveal the coupling of electrons with phonons at energies Ω_1_ = 177 and Ω_2_ = 54 meV, and Ω_3_ = 82 meV (gray arrow).

The high-resolution ARPES data allow us to resolve the electron-phonon coupling-induced self-energy effects in the folded Dirac cones at the Γ point. Figure 2a shows the three-dimensional band structure measured by the laser source with sharp dispersions, and a kink (indicated by the red arrow) is observed in the dispersion along the Γ–K^*^ (Γ–M) direction (represented with a red dashed line in Fig. 2a) for the α pocket. The kink is more clearly resolved in the dispersion image in Fig. 2b. By fitting the momentum distribution curves (MDCs) in Fig. 2c, we extract the dispersion (black curve in Fig. 2d) and the peak width, which can be converted into ReΣ and |ImΣ| using standard ARPES analysis [13]. We extract ReΣ by assuming a linear bare band dispersion (dotted line in Fig. 2d). A peak at −Ω_1_ (red arrow in Fig. 2e) and a shoulder at −Ω_2_ (black arrow) is observed, which is accompanied by an increase in the scattering rate in |ImΣ| (Fig. 2f), and a possible coupling to an additional phonon at Ω_3_ (gray arrow) is also observed. Further fitting of the Eliashberg function gives phonon energies of Ω_1_ = 177 ± 1 meV and Ω_2_ = 54 ± 4 meV (see the detailed analysis in Fig. S4 of the online supplementary material). We note that electron-phonon coupling has been reported and suggested as important for superconducting CaC_6_ [17,18] or Li-decorated graphene samples [19,45]. Here, the high data quality of the laser source allows us to resolve fine structures in the self-energy, indicating coupling of electrons to multiple phonons. Such mode-specific electron-phonon interaction lays an important foundation for further investigating the role of these phonons in the relaxation dynamics.

**Figure 3.**
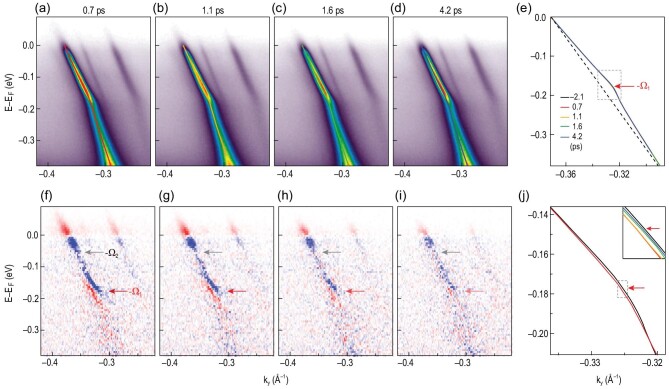
TrARPES dispersion images measured at different delay times after pumping at a pump fluence of 215 μJ/cm^2^. (a)–(d) Evolution of the dispersion images measured at different delay times. (e) Extracted dispersion at different delay times. (f)–(i) Differential images obtained by subtracting dispersion images measured at −2.1 ps from (a)–(d). Red and blue pixels respectively represent the increase and decrease in intensity. Red and gray arrows indicate the threshold effect at −Ω_1_ and −Ω_2_ energies. (j) Enlargement of the extracted dispersions (indicated by the gray dotted box in (e)) to show a comparison between the dispersions at 0.7 ps (red curve) and −2.1 ps (black curve). The inset displays the enlarged dispersion (over the gray dotted box in (j)) at different delay times to show the dynamic evolution of the kink. Red arrows indicate an energy of −Ω_1_.

The electron dynamics is revealed by comparing dispersion images measured at different delay times with a pump photon energy of 1.55 eV at a pump fluence of 215 μJ/cm^2^. Figure 3a–d shows dispersion images measured at 0.7, 1.1, 1.6 and 4.2 ps after pump excitation, respectively, and the extracted dispersions at different delay times are plotted in Fig. 3e. After subtracting the dispersion image measured at -2.1 ps, the spectral weight redistribution is clearly resolved in the differential images shown in Fig. 3f–i. An increase in intensity above *E*_*F*_ is clearly resolved (highlighted in red), indicating photo-excited electrons above *E*_*F*_. In addition, a suppression of intensity below *E*_*F*_ indicates photo-excited holes below *E*_*F*_ (highlighted in blue). In contrast to previous TrARPES studies where the TrARPES signal was widely spread out in a large energy range of approximately 1 eV [23–30,32], our TrARPES signal is mostly confined in a much smaller energy range within 177 meV (indicated by red arrows) with a much stronger TrARPES signal observed

within 54 meV (gray arrows), indicating the energy threshold effect in the TrARPES signal and the relaxation dynamics. Such an energy threshold effect with TrARPES signal confined within 177 meV is ubiquitous across the entire BZ (for more data in a larger momentum space, see Fig. S5 of the online supplementary material). In addition, the differential images in Fig. 3f–i show an unusual fine feature indicated by red arrows around −Ω_1_ with an increase in intensity (highlighted in red) at the negative side of the peak and a decrease in intensity at the positive side (highlighted in blue), indicating a modification to the dispersions measured at different delay times. The enlarged dispersions in Fig. 3j further reveal the dynamical modification of the dispersion near the kink energy at different delay times (see Fig. S6 of the online supplementary material for a detailed analysis of the self-energy at −2.1 ps and 0.7 ps). At later delay times, the dispersion almost recovers (see the comparison between the blue curve at 4.2 ps and the black curve at −2.1 ps in the inset of Fig. 3j).

**Figure 4.**
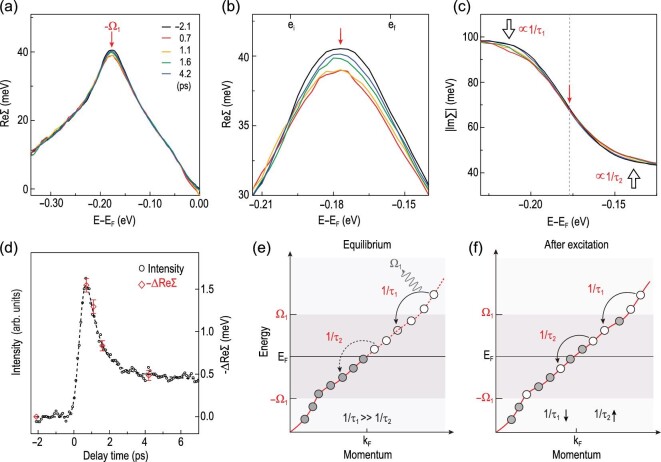
Self-energy dynamics and carrier relaxation dynamics. (a) Extracted ReΣ at different delay times. (b) Enlargement of ReΣ to show the dynamical change of ReΣ around −Ω_1_ (red arrow). (c) Extracted |ImΣ| at different delay times to show the renormalization of the scattering rate around −Ω_1_ after pump excitation, as indicated by black open arrows. (d) A comparison between the pump-induced decrease in −ReΣ as a function of the delay time (red symbols, obtained by averaging −ReΣ over the energy range (e_*i*_ to e_*f*_) indicated by black short marks in (b)) and the pump-induced population (black symbols and dotted curve) obtained by integrating from 0 to 50 meV above the Fermi energy. (e) and (f) Schematics of the electron redistribution after pump excitation and the related renormalization of the scattering rate 1/τ for holes (or electrons) inside and outside the phonon window, which gives the dynamical change of the self-energy around the phonon energy. Gray and white circles represent electrons and holes, respectively.

To further reveal the underlying physics behind the dynamical change of the dispersion in the time domain, we show in Fig. 4 an analysis of ReΣ and |ImΣ| at different delay times. A decrease in the peak is observed in ReΣ in Fig. 4a and in the enlarged ReΣ near the kink energy in Fig. 4b, which gradually recovers at a later delay time (from orange to blue curves in Fig. 4b). A corresponding change is also observed in |ImΣ| (see the black open arrows in Fig. 4c), which is related to ReΣ by the Kramers–Kronig relationship (see Fig. S7 of the online supplementary material for more details about the dynamical change of self-energy at difference delay times). To check if the electron-phonon coupling-induced self-energy effect is correlated with the pump-induced spectral weight transfer revealed in Fig. 3f–i, we show in Fig. 4d a comparison between the temporal evolution of the pump-induced change in the self-energy −ΔReΣ (red symbol) and the electron population above *E*_*F*_ that is obtained by integrating the TrARPES intensity from 0 to 50 meV (black symbols and dotted curve). The same temporal evolution suggests a correlation between the dynamical self-energy and the pump-induced spectral weight redistribution and the corresponding change in the scattering phase space [46,47]. In the equilibrium state (Fig. 4e), the scattering rate for electrons (holes) inside the phonon energy window (±Ω_1_, 0), 1/τ_2_, is much less than that outside this window, 1/τ_1_, due to insufficient energy to emit a phonon at Ω_1_, as indicated by the jump in |ImΣ| (Fig. 2f). Upon pump excitation, electrons are populated above the Fermi energy *E*_*F*_ and holes below *E*_*F*_ (indicated by gray and white circles on the red curve in Fig. 4f); therefore, the scattering rate for holes (or electrons) inside the phonon window (1/τ_2_) increases due to an increase in the scattering phase space to scatter into, while outside the phonon window (1/τ_1_) it decreases. Such a change in the scattering rate by the photon-induced spectral weight redistribution leads to a dynamical modification of |ImΣ| in Fig. 4c and ReΣ in Fig. 4b, implying the significant role of the phonons that are coupled to electrons. We note that a dynamical change in the self-energy has been reported in a high-temperature BSCCO superconductor, especially in the superconducting state [48–50]. Here we report the first observation of dynamical change in the self-energy in a non-superconducting material.

**Figure 5.**
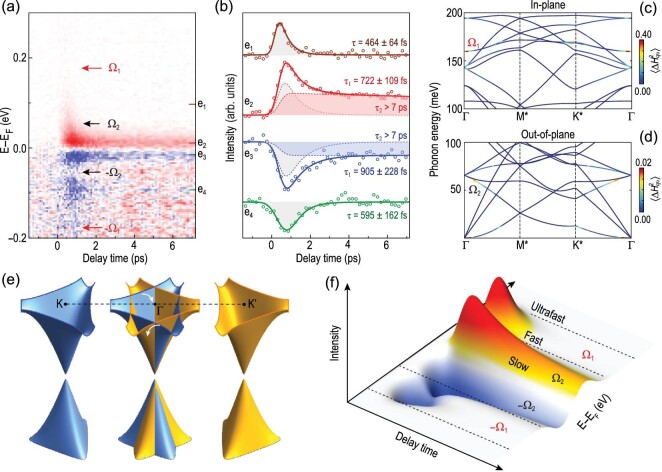
Phonon threshold effect and hierarchical relaxation of electrons in different energy windows. (a) Evolution of momentum-integrated differential intensity with energy and delay time. Red and blue pixels respectively represent the increase and decrease in intensity. (b) Differential intensity as a function of the delay time at energies indicated by colored tick marks in (a). Solid curves are fitting results. Gray shaded regions in e_1_–e_4_ indicate the fast relaxation, while red and blue shaded regions in e_2_ and e_3_ represent the slow relaxation component. (c) and (d) Calculated phonon dispersion and electron-phonon coupling strength of Li-intercalated graphene for in-plane and out-of-plane phonon modes, respectively. (e) Schematics of electron-phonon coupling in the Li-intercalated graphene. (f) Schematic of the phonon threshold effect with hierarchical relaxation.

The electron-phonon interaction not only modifies the self-energy in the time domain but also sets energy thresholds, as indicated by the red and gray arrows in Fig. 3f–i, and its relation to the relaxation dynamics is further revealed in Fig. 5. The temporal evolution as a function of energy and delay time (Fig. 5a) and the selected curves at different energies (Fig. 5b) reveal hierarchical relaxation times in different energy windows defined by the two strongly coupled phonons, which are summarized below: (1) for energy windows ±(∞, Ω_1_) in which photo-excited carriers have sufficient energy to emit phonons with energies of Ω_1_ and Ω_2_, the relaxation is ‘ultrafast’—faster than 337 fs (see Fig. S8 of the online supplementary material for a detailed analysis of energy-dependent relaxation time) and there is negligible TrARPES signal; (2) for energy windows ±(Ω_1_, Ω_2_) in which photo-excited carriers can emit phonons at Ω_2_ but not Ω_1_, the relaxation is ‘fast’, within a few hundred femtoseconds (curves at e_1_ and e_4_ in Fig. 5b); (3) for energy windows ±(Ω_2_, 0) in which the relaxation requires involvement of acoustic phonons at even lower energy, the relaxation is ‘slow’ with an additional component persisting beyond 7 ps (highlighted as red and blue shaded regions for curves e_2_ and e_3_ in Fig. 5b) that involves relaxation through another mechanism, e.g. acoustic phonons. The observation of distinct relaxation time scales in different energy regimes ±(∞, Ω_1_), ±(Ω_1_, Ω_2_) and ±(Ω_2_, 0) establishes a direct correlation between the hierarchical relaxation times and the two strongly coupled phonons at ±(Ω_1_, 0) and ±(Ω_2_, 0). Therefore, our results show that the coupled phonons at Ω_1_ and Ω_2_ play a dominant role in the relaxation of electrons in graphene. Theoretical calculations of the phonon dispersion and electron-phonon coupling strength for the Li-intercalated graphene (see the online supplementary material for details of the calculation and Fig. S9 therein for more data) have identified that the two phonons at Ω_1_ and Ω_2_ that are coupled to electrons and thereby dominate the relaxation dynamics are the in-plane TO phonon *A*_1*g*_ (Fig. 5c) and the out-of-plane ZA phonon (Fig. 5d).

## CONCLUSION

To summarize, by strategically folding the Dirac cones to Γ (Fig. 5e), high-resolution TrARPES measurements allow us to visualize the coupling of electrons to two strongly coupled phonon modes in the time domain. The coupling of electrons with multiple phonons sets energy windows for electron relaxation with hierarchical relaxation dynamics, as schematically illustrated in Fig. 5f. Moreover, the change in the electron self-energy suggests a dynamical modification of coupling between electrons and phonons, and provides important information for considering the non-equilibrium electron-boson interactions in other systems. Our work not only provides direct experimental evidence for the dominant role of mode-specific phonons in the relaxation dynamics of Kekulé-ordered graphene, but also provides a new material platform for exploring the engineering of Dirac cones by light-matter interaction.

## METHODS

### Sample preparation

Bilayer graphene was grown by flash annealing the Si face of 6H-SiC(0001) substrates in ultrahigh vacuum. Lithium intercalation was performed by *in situ* deposition of Li from an alkali metal dispenser (SAES), with the graphene sample maintained at 320 K [43]. The intercalation process was monitored by low-energy electron diffraction and ARPES measurements. The intercalation releases the buffer layer [44] underneath the bilayer graphene, eventually resulting in Kekulé-ordered trilayer graphene with Li atoms inserted between the graphene layers.

### TrARPES measurements

TrARPES measurements were performed in the home laboratory at Tsinghua University at 80 K in a working vacuum better than 6 × 10^−11^ Torr. The pump photon energy was 1.55 eV and the pump fluence was set to 215 μJ/cm^2^. A pulsed laser source at 6.2 eV with a repetition rate of 3.8 MHz was used as the probe source. The overall time resolution was set to 480 fs. The Fermi edge of the graphene sample measured at 80 K showed an energy width of 33 meV, from which the overall instrumental energy resolution was extracted to be 16 meV after removing the thermal broadening (for more details, see Fig. S1 of the online supplementary material). Moreover, the reduction of photon energy compared to HHG also leads to major improvement in the momentum resolution. Since the momentum resolution at the Fermi energy *E*_*F*_ is $\Delta k \propto \sqrt{h\nu -\phi }$, where *h*ν and φ ≈ 4.3 eV are the photon energy and work function, respectively, the reduction in photon energy from *h*ν ≥ 25 to 6.2 eV leads to at least a 3 times improvement in Δ*k*, with an ultimate experimental resolution of Δ*k* = 0.001 Å^−1^. The greatly improved energy and momentum resolution together with the high data acquisition efficiency are critical to the successful observation of the electron-phonon coupling-induced kink in the TrARPES data and the phonon threshold effect.

## Supplementary Material

nwab175_Supplemental_File
