# Supplementary material for: Self-energy dynamics and the mode-specific phonon threshold effect in Kekulé-ordered graphene
Source: Natl Sci Rev. 2021 Sep 16;9(5):nwab175. doi: 10.1093/nsr/nwab175 (PMC9155635; doi:10.1093/nsr/nwab175)
Supplement: nwab175_Supplemental_File [file nwab175_supplemental_file.pdf]

Supplementary information — Self-energy dynamics and mode-specific phonon threshold effect in a Kekulé-ordered graphene

Hongyun Zhang[1†], Changhua Bao[1†], Michael Schüler[2], Shaohua Zhou[1], Qian Li[1], Laipeng Luo[1], Wei Yao[1], Zhong Wang[3], Thomas P. Devereaux[2,4] & Shuyun Zhou[1,5*]

[1]*State Key Laboratory of Low Dimensional Quantum Physics and Department of Physics, Tsinghua University, Beijing 100084, P. R. China*

[2]*Stanford Institute for Materials and Energy Sciences (SIMES), SLAC National Accelerator Laboratory, Menlo Park, California 94025, USA*

[3]*Institute for Advanced Study, Tsinghua University, Beijing, 100084, P. R. China*

[4]*Department of Materials Science and Engineering, Stanford University, Stanford, CA 94035, USA*

[5]*Frontier Science Center for Quantum Information, Beijing 100084, P. R. China*

† *These authors contributed equally to this work.*

* *Correspondence should be sent to syzhou@mail.tsinghua.edu.cn.*

Li intercalation of bilayer graphene and release of buffer layer. Figure S1 shows the schematic illustration of bilayer graphene before Li intercalation (left panel), which has a buffer layer bonded to the SiC substrate and does not contribute to the π band. The intercalation releases the bonding between the buffer layer and the SiC substrate, resulting in a Li-intercalated trilayer graphene with Kekulé order as schematically shown in the right panel.

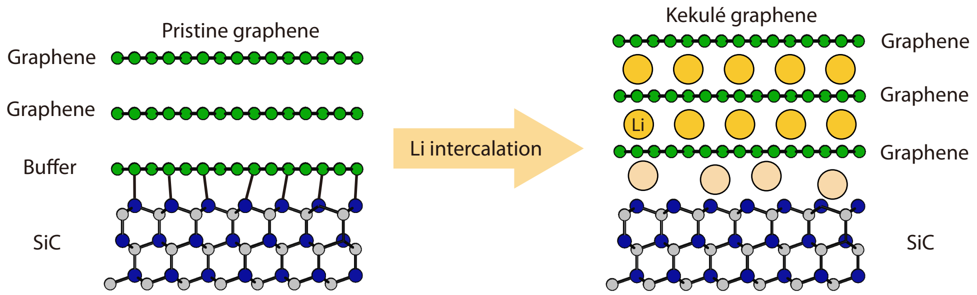

Figure S1: **Schematic illustration of Li intercalation of bilayer graphene.** Schematic illustration of bilayer graphene on a buffer layer before Li intercalation (left), and the Li-intercalated trilayer graphene with Kekulé order (right).

Fermi surface map and dispersion image of AA-stacked trilayer graphene with Kekulé order. Figure S2a shows the Fermi surface map of the trilayer Kekulé-ordered graphene, which clearly shows the three pockets with different size and the replica bands around the Γ point. Figure S2b shows the dispersion image measured along K-M direction as indicated by red solid line in Fig S2a. The dispersion image shows the high electron doping with the Dirac point energy at -2.14 eV of the largest pocket. The Fermi surface map as well as the dispersion image together suggest a AA stacking of this trilayer Kekulé-ordered graphene.

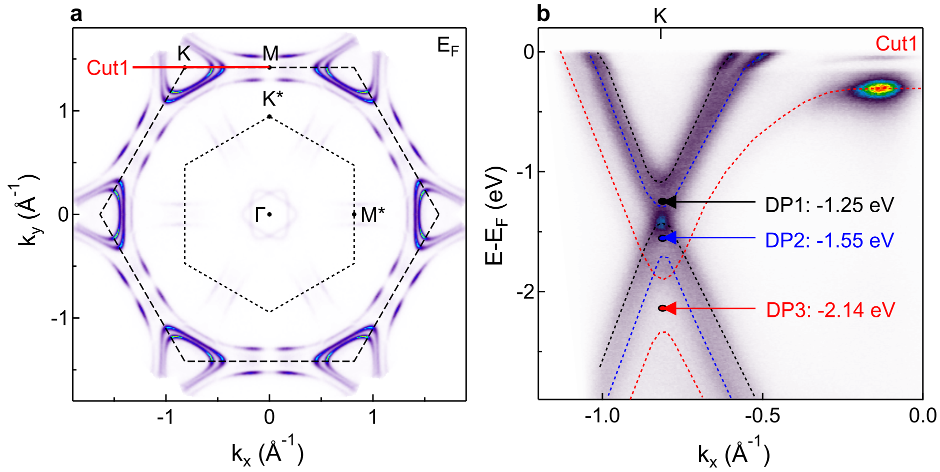

Figure S2: **Fermi surface map and dispersion image of AA-stacked trilayer graphene with Kekulé order.** **a**, Fermi surface map of the Kekulé-ordered trilayer graphene. **b**, Dispersion image measured along K-M direction as indicated by the red solid line in **a**, with the Dirac point energy of each Dirac cone labeled.

Energy and time resolution. The Fermi edge measured from the graphene at 80 K shows an energy width of 33 $\pm$ 1 meV by fitting with the Fermi-Dirac distribution function (Fig. S3a). Therefore, the overall instrumental energy resolution is extracted to be $\sqrt{33^2 - 29^2} \approx 16$ meV after removing the thermal broadening of 29 meV at 80 K. The time resolution is determined by the cross correlation between pump and probe laser pulses, which is reflected from the rising edge of the measured TrARPES trace (shown by red symbols in Fig. S3b). The TrARPES data is fitting by a Gaussian function convolved with the product of the step function and a single-exponential function,

$$I(t) = A(1 + erf(\frac{t - t_0}{\Delta t} - \frac{\Delta t}{2\tau}))e^{-\frac{t-t_0}{\tau}} + B$$

where Δt is the width of the rising edge and τ is the relaxation time. Therefore, the extracted time resolution is determined to be 480 $\pm$ 70 fs.

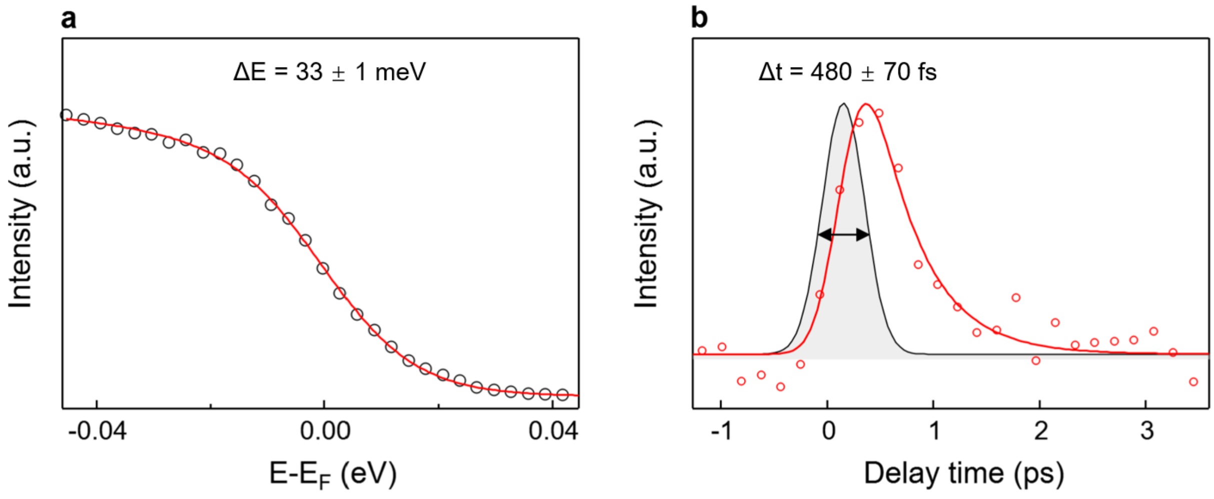

Figure S3: **Experimental energy and time resolution. a**, Fitting of the Fermi edge measured on the sample at 80 K with an energy of 33 meV. **b**, Fitting of TrARPES intensity curve (red marks and red curve) gives a time resolution of 480 $\pm$ 70 fs.

Determination of coupled phonon energies by extraction of Eliashberg function. By fitting ReΣ with the Eliashberg function[1], we can determine the coupled phonon energies from the experimental results. Broken curves in Fig. S4a are components of the extracted Eliashberg function, which show two obvious peaks at energy of -54 and -177 meV (black broken curves) and a broad background (gray). These two phonons also correspond to those observed in ImΣ (Fig. S4c), with an additional jump at -82 meV (gray arrow), indicating possible coupling to a third phonon. Fitting ReΣ along the Γ-M* (Γ-K) direction (Fig. S4b) also gives similar results, with peak energies at -56 and -173 meV and corresponding feature in ImΣ as shown in Fig. S4d. Figure S4e,f shows the calculated ReΣ and ImΣ taking into account the coupling of electrons to the in-plane and out-of-plane phonon modes (indicated by red and black arrows).

Assuming intraband scattering, the retarded electron-phonon self-energy within the non-selfconsistent Migdal approximation is computed [2] according to

$$\Sigma_\alpha(\vec{k},\omega) = \frac{1}{2N_k}\sum_{\vec{q}\nu}\sum_{\alpha'}\left|g^{\nu}_{\alpha\alpha'}(\vec{k},\vec{q})\right|^2\left[\frac{n_{\vec{q}\nu}+1-f_{\vec{k}-\vec{q}\alpha'}}{\omega-\varepsilon_{\alpha'}(\vec{k}-\vec{q})-\omega_{\vec{q}\nu}+i\eta}+\frac{n_{\vec{q}\nu}+f_{\vec{k}-\vec{q}\alpha'}}{\omega-\varepsilon_{\alpha'}(\vec{k}-\vec{q})+\omega_{\vec{q}\nu}+i\eta}\right] \quad (1)$$

Here, η is an infinitesimally small positive number (we fix $\eta = 10^{-4}$ a.u.), $f_{\vec{k}\alpha}$ denotes occupation of the Bloch state $|\psi_{\vec{k}\alpha}\rangle$, while $n_{\vec{q}\nu}$ is the occupation of corresponding phonon mode (given by the Bose distribution). We evaluated eq. (1) for $T = 80$ K in accordance with the experiment. Note that the electron-phonon matrix elements $g^{\nu}_{\alpha\alpha'}(\vec{k},\vec{q})$ are transformed to the Bloch basis. We used a $N_k = 400 \times 400$ sampling of the Brillouin zone, ensuring convergence.

The calculation results agree very well with the experimental data assuming a zero value of ReΣ at the Fermi energy. Test calculations excluding out-of-plane coupling reveal that the feature denoted by the black arrow disappears, thus confirming that Ω_2 is an out-of-plane mode.

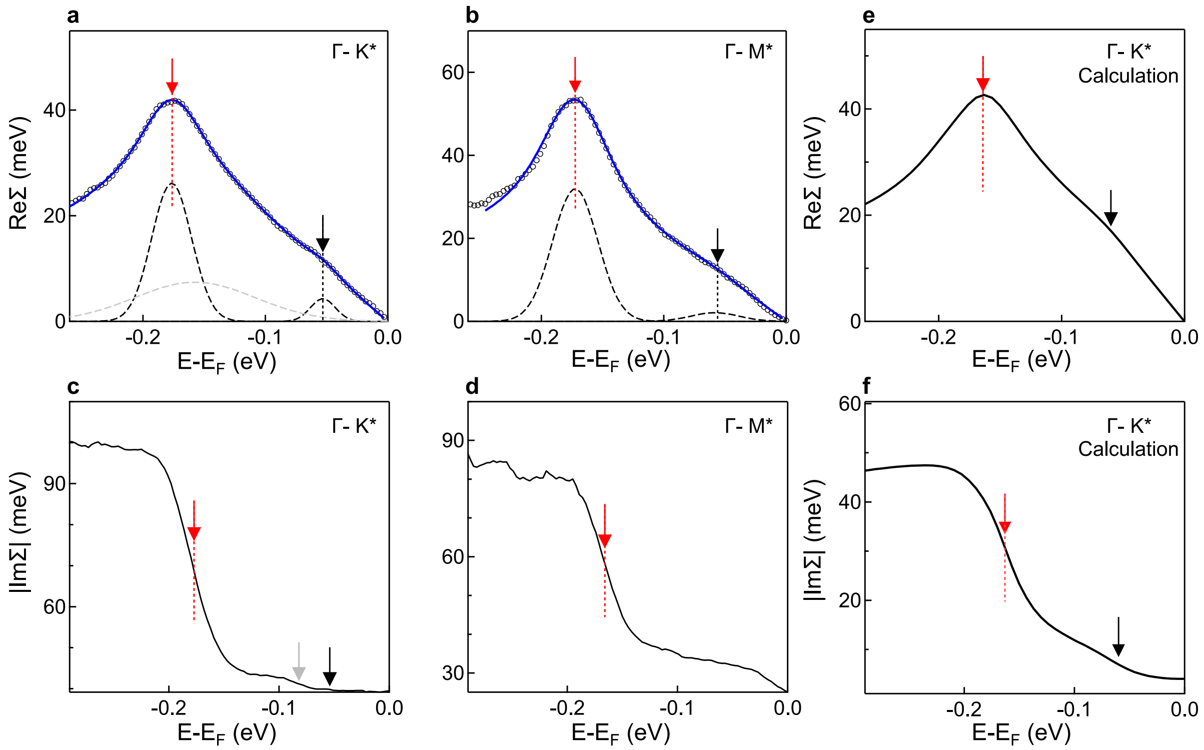

Figure S4: **Determination of coupled phonon energies by extraction of Eliashberg function.** **a,b**, ReΣ (markers) from the dispersion along Γ-K* (Γ-M) and Γ-M* (Γ-K) direction, respectively. Blue curves are fitting results to extract the Eliashberg function, with the components of Eliashberg function plotted by broken curves. **c,d**, Corresponding ImΣ, which shows jumps at phonon energy indicated by colored arrows. **e,f**, Calculated ReΣ and ImΣ by coupling electrons to the in-plane and out-of-plane phonons revealed in Fig. S9 (colored arrows).

Phonon threshold effect in the momentum space. The phonon threshold effect is also observed in other regions of the momentum space. Figure S5b shows the differential image along Γ-M* direction at delay time of 700 fs. Phonon-window effect at -177 meV (red arrow in Fig. S5b) is also clearly observed. Indeed, such phonon threshold effect is also observed in the entire momentum space measured (see intensity maps in Fig. S5c-n), with strong differential signal within -177 meV, and negligible signal beyond the phonon-window.

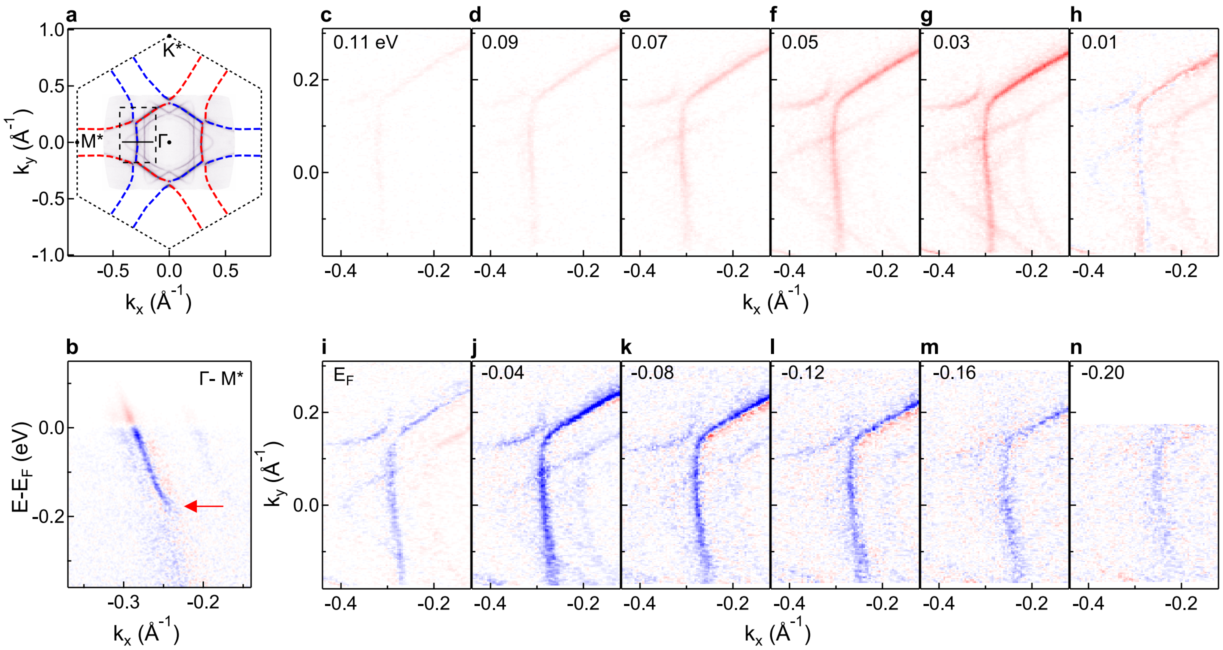

Figure S5: **Phonon threshold effect in the entire momentum space.** **a**, Fermi surface map. The cut along Γ-M* direction for data shown in **b** is indicated by black solid line. **b**, Differential image measured along the Γ-M* direction at delay time of 700 fs. Red arrow indicates the phonon threshold effect at -177 meV. **c-n**, TrARPES differential intensity maps at delay time of 700 fs at different energies for momentum regions marked by broken rectangle in **a**.

Dynamic evolution of the kink at -Ω_1 upon pump excitation. Figure S6 shows more analysis to reveal the dynamic evolution of the kink. The differential image in Fig. S6c suggests that there is a shift of dispersion near the kink energy (pointed by red arrow). This is further confirmed by the extracted dispersion (Fig. S6d) and in particular the zoomed-in dispersion (Fig. S6e). Such change in the dispersion near the kink energy is confirmed by the MDCs shown in Fig. S6f, and results in a reduction of the peak amplitude in ReΣ (Fig. S6h) and broadening of FWHM (Fig. S6g). We note that similar change in the self-energy has been reported in BSCCO[3,4] as ultrafast quenching of electron-phonon interaction in the superconducting state upon laser excitation[3]. Our Li-intercalated graphene is unlikely superconducting at the measurement temperature of 80 K. Therefore, the observed apparent shift of the kink is interpreted as phonon threshold induced change in the self-energy due to dynamical spectral weight transfer.

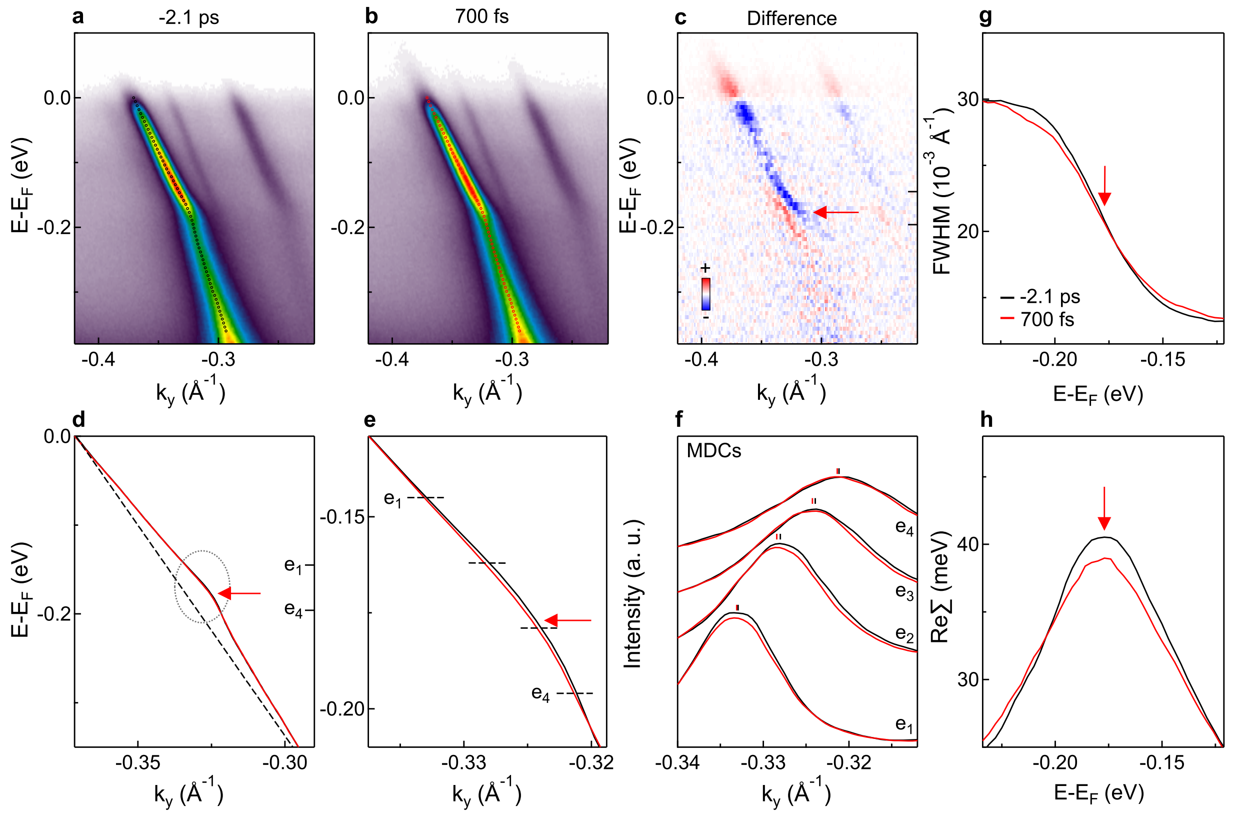

Figure S6: **Dynamic evolution of the kink upon pump excitation. a,b**, Dispersion images along the Γ-K* (Γ-M) direction at delay time of -2.1 ps and 700 fs, respectively. **c**, Differential image by subtracting **a** from **b**. **d**, Extracted dispersion at -2.1 ps (black curve) and 700 fs (red curve). Black broken line indicates the bare band dispersion. **e**, Zoomed-in dispersion near the kink energy to show the pump induced change in the dispersion around -177 meV. **f**, MDCs at energy from e_1 to e_4 indicated in **e**. Black and red tick marks indicate the peak positions. **g,h**, FWHM and ReΣ at -2.1 ps (black) and 0.7 ps (red).

Difference curves of ReΣ and |ImΣ| at different delay times. To better resolve the dynamical change of the self-energy with changing of delay time, we show in Fig. S7 the difference curves of ReΣ and |ImΣ| at different delay times, which are obtained by subtracting the self-energy extracted at -2.1 ps from that at different delay times. The differential ReΣ clearly shows that the real part of the self-energy decreases at 0.7 ps and then gradually recover with increasing of delay time, while the |ImΣ| shows a broadening behavior at 0.7 ps and gradually recover.

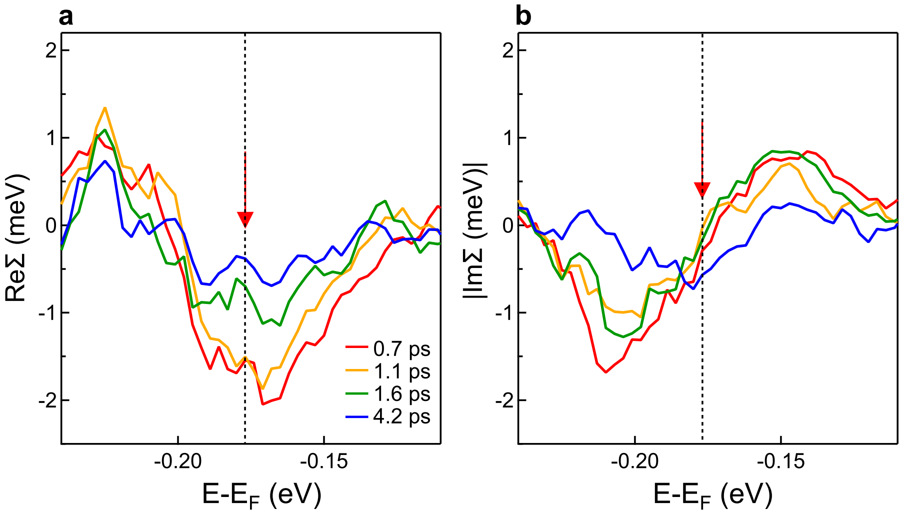

Figure S7: **Difference curves of ReΣ and |ImΣ| at different delay times. a,b**, Difference curves of ReΣ and |ImΣ| at different delay times respectively. Red arrows indicate the phonon energy of Ω_1=-177 meV.

Hierarchical relaxation and more data on the relaxation time. Figure S8a is a plot of the evolution of momentum-integrated differential intensity with energy and delay time, which only shows differential signal within energy of Ω_1, indicating the energy threshold between "ultrafast" and "fast" regime. Figure S8b shows the relaxation curves at different energy regimes. The relaxation curve integrated over 177-300 meV in the "ultrafast" regime is shown by blue dotted curve, from which electrons relax within the time resolution and leads to a Gaussian-shaped peak. The relaxation in the "fast" regime is shown by yellow dotted curves, and the relaxation time of energy range between 78 and 177 meV is 337 fs, which suggests that the relaxation time in the "ultrafast" regime should be even faster than 337 fs. For electrons in the "slow" regime, the relaxation curves (red dotted curve) show an additional component (red shading area) with relaxation time larger than 7 ps, which clearly sets the energy threshold between the "fast" and "slow" regime. The extracted relaxation time for τ_1 and τ_2 are plotted as black and red symbols in Fig. S8c.

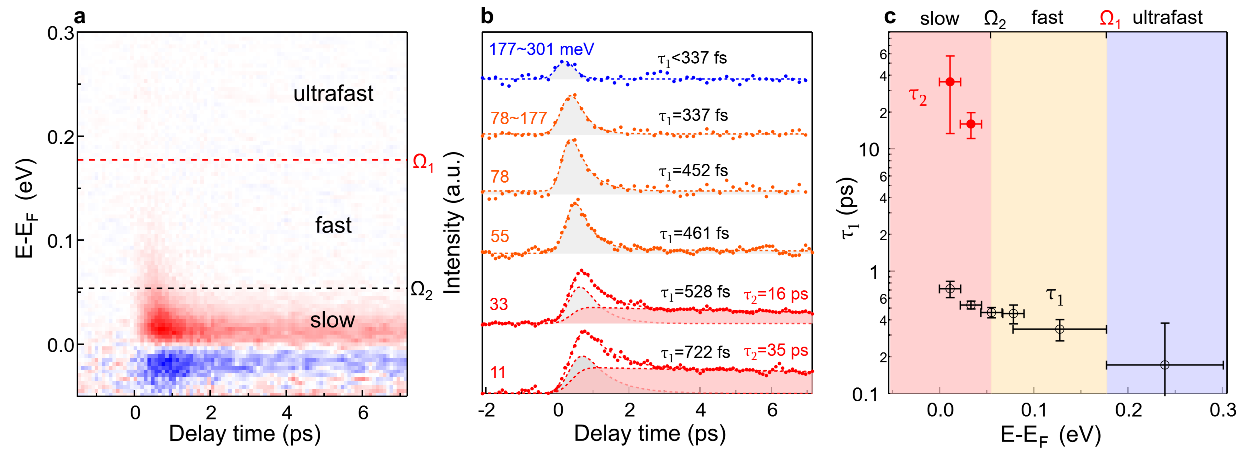

Figure S8: **Hierarchical relaxation at different energy regimes.** **a**, Evolution of momentum-integrated differential intensity with energy and delay time. Red and blue colors represent increase and decrease of intensity, respectively. **b**, Relaxation curves (colored symbols) and fitting results (colored dotted curves) at different energies. **c**, Extracted relaxation time of the faster component τ_1 (black symbols) and the slower component τ_2 (red symbols) as a function of energy.

Calculation of phonon spectra and determination of phonon modes. The ARPES signal of the largest pocket mostly comes from the bottom graphene layer due to the high doping and the effective decoupling by Li intercalation. Therefore, we consider a single layer of graphene with Kekulé order as a simple model, which shows very good agreement to the experimental data. We employ atomic units (a.u.) unless stated otherwise.

The Hamiltonian of the systems comprises the electron (e), the electron-phonon coupling (e-ph) and the phonon Hamiltonian:

$$\hat{H} = \hat{H}_{\mathrm{e}} + \hat{H}_{\mathrm{e-ph}} + \hat{H}_{\mathrm{ph}} \ . \tag{2}$$

We describe the Hamiltonian for the π electrons by nearest-neighbor tight-binding model with hopping constant $J = 2.8$ eV for graphene, while we assume Kekulé-O order[5] with hopping constants $J_1 = 2.83$ eV, $J_2 = 3.02$ eV for Kekulé-ordered graphene.

For obtaining a model for the phonon modes in the graphene and Kekulé-ordered graphene, we employed the force constant model[6], which yields excellent agreement with experiments on graphite [7]. We computed the phonon dispersion $\omega_{\vec{q}\nu}$ and the corresponding mode eigenvectors $\vec{e}_{\vec{q}\nu,m}$ for atom m in the unit cell for monolayer graphene both in the graphene and in the Kekulé unit cell (shown in Fig. 1a in the main text). This determines the phonon Hamiltonian $\hat{H}_{\mathrm{ph}} = \sum_{\vec{q}\nu} \omega_{\vec{q}\nu} \hat{b}^{\dagger}_{\vec{q}\nu} \hat{b}_{\vec{q}\nu}$ ($\hat{b}_{\vec{q}\nu}$ is the phonon annihilation operator with respect to momentum $\vec{q}$ and mode ν).

The electron-phonon coupling Hamiltonian has the generic form

$$\hat{H}_{\mathrm{e-ph}} = \frac{1}{\sqrt{N_k}} \sum_{\vec{k},mm'} \sum_{\vec{q}\nu} g^{\nu}_{mm'}(\vec{k},\vec{q}) \hat{c}^{\dagger}_{\vec{k}+\vec{q}m} \hat{c}_{\vec{k}m'} \hat{X}_{\vec{q}\nu} \ , \tag{3}$$

where $\hat{c}^{\dagger}_{\vec{k}m}$ ($\hat{c}_{\vec{k}m}$) is the electron creation (annihilation) operator with respect to the sublattice site m, while

$\hat{X}_{\vec{q}\nu} = (\hat{b}^{\dagger}_{-\vec{q}\nu} + \hat{b}_{\vec{q}\nu})/\sqrt{2}$. The electron-phonon coupling matrix element is defined as

$$g^{\nu}_{mm'}(\vec{k},\vec{q}) = \sqrt{\omega_{\vec{q}\nu}}\langle \vec{k}+\vec{q}m|\Delta\hat{H}_{\vec{q}\nu}|\vec{k}m'\rangle \ , \tag{4}$$

where $\Delta\hat{H}_{\vec{q}\nu}$ denotes the change of the electronic Hamiltonian upon atom displacements associated to mode $(\vec{q}\nu)$. For the electron-phonon Hamiltonian $\Delta\hat{H}_{\vec{q}\nu}$ we consider two mechanisms: (i) modulation of hopping (deformation potential), (ii) dipole coupling to an internal electric field. Mechanism (i) gives rise to coupling of the in-plane phonon modes to the electrons, while (ii) affects out-of-plane modes. Direct (linear) coupling to out-of-plane modes is excluded for free-standing graphene due to mirror symmetry; in a heterostructure, however, the coupling becomes finite.

The deformation potential contribution can be extracted from the tight-binding model [8]. The only parameter is the relative variation of the hopping upon atom movement: $q_0 = (1/J)\partial J/\partial b$ (b is the distance between two carbon atoms). A straightforward derivation yields

$$\begin{aligned}\langle \vec{k}+\vec{q}m|\Delta\hat{H}_{\vec{q}\nu}|\vec{k}m'\rangle = {} & \frac{q_0}{(M\omega^2_{\vec{q}\nu})^{1/2}}\sum_{\vec{L}} e^{i\vec{k}\cdot\vec{L}} J_{\vec{L}}\vec{v}_{mm'}(\vec{L})\cdot\vec{e}_{\vec{q}\nu,m} \\ & - \frac{q_0}{(M\omega^2_{\vec{q}\nu})^{1/2}}\sum_{\vec{L}} e^{i(\vec{k}+\vec{q})\cdot\vec{L}} J_{\vec{L}}\vec{v}_{mm'}(\vec{L})\cdot\vec{e}_{\vec{q}\nu,m'} \ .\end{aligned} \tag{5}$$

Here, $\vec{v}_{mm'}(\vec{L})$ denotes the vector connecting atom m in the unit cell and atom m' in the same ($\vec{L}=0$) or neighboring ($\vec{L}\neq 0$) unit cell. The sum over $\vec{L}$ includes all lattice vectors such that $|\vec{v}_{mm'}(\vec{L})| = a_{\mathrm{CC}}$ (a_{CC} is the distance between two neighboring carbon atoms). For graphene $J_{\vec{L}} = J$, while $J_{\vec{L}} = J_1$ or $J_{\vec{L}} = J_2$ for Kekulé-ordered graphene.

For modeling the interaction of electrons with the out-of-plane modes, we consider each Li atom in the heterostructure as a point charge giving rise to an electric field. The effective field in the z direction E_0 is identical for each carbon atom, and is treated as a parameter. The coupling matrix element becomes

$$\langle \vec{k}+\vec{q}m|\Delta\hat{H}_{\vec{q}\nu}|\vec{k}m'\rangle = -\delta_{mm'}\frac{E_0}{(M\omega^2_{\vec{q}\nu})^{1/2}}[\vec{e}_{\vec{q}\nu,m}]_z \ . \tag{6}$$

To determine the contribution of the specific phonon modes in the electron-phonon coupling of electrons at the Fermi level (E_F), we computed the averaged coupling

$$\langle \Delta \hat{H}^2_{\vec{q}\nu} \rangle = \frac{2}{\rho(E_F) N_k} \sum_{\vec{k}\alpha} \left| \langle \psi_{\vec{k}+\vec{q}\alpha} | \Delta \hat{H}_{\vec{q}\nu} | \psi_{\vec{k}\alpha} \rangle \right|^2 \delta\left(\varepsilon_\alpha(\vec{k}) - E_F \right) \delta\left(\varepsilon_\alpha(\vec{k} - \vec{q}) - \omega_{\vec{q}\nu} - \varepsilon_\alpha(\vec{k}) \right) \ . \quad (7)$$

Here, $|\psi_{\vec{k}\alpha}\rangle$ stand for the Bloch states with energy $\varepsilon_\alpha(\vec{k})$; $\rho(\varepsilon)$ denotes the density of states.

We fixed $q_0 = 0.1$ a.u. and $E_0 = 0.03$ a.u. by comparing to resulting photoemission spectra to the experiment. For graphene, this gives rise to predominant in-plane coupling of the LO and TO mode near Γ and the degenerate LA and LO mode at K (Fig. S9a), while the out-of-plane mode ZA mode near K couples strongest (Fig. S9c). For Kekulé-ordered graphene (Fig. S9b,d), the TO mode folded from K (extended Brillouin zone) onto Γ dominates the in-plane electron-phonon coupling (labelled as Ω_1). The calculated electron-phonon coupling strength is roughly twice as big as the strongest coupling in regular graphene. Similarly, we find that the out-of-plane phonon mode originating from the ZA mode folded to Γ (labelled as Ω_2 in Fig. S9d) strongly couples to the electrons.

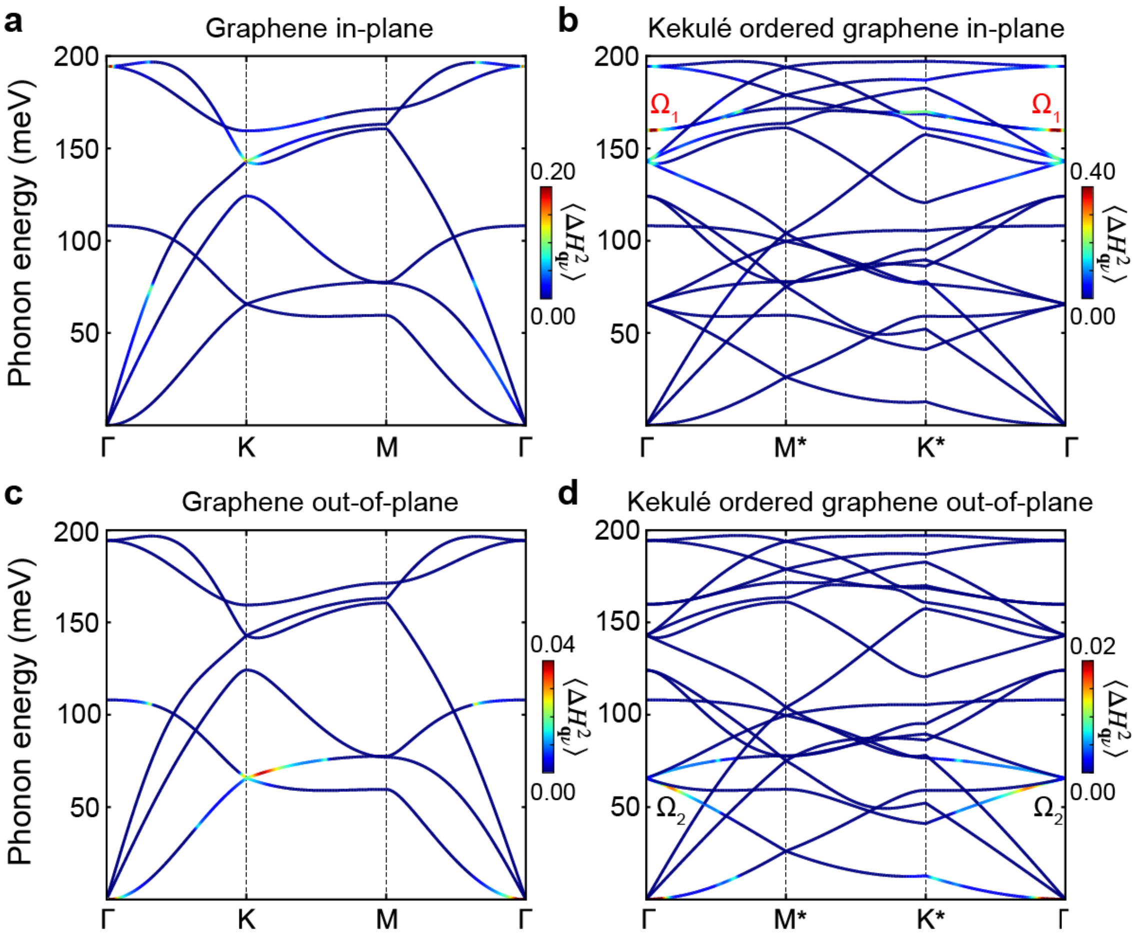

Figure S9: **Calculation of phonon dispersion and electron-phonon coupling strength. a-d**, Calculated in-plane and out-of plane phonon dispersion and electron-phonon coupling strength for graphene and Kekulé-ordered graphene, respectively. Different color represents the calculated electron-phonon coupling strength.

1. Yang, S.-L. *et al.* Superconducting graphene sheets in CaC_6 enabled by phonon-mediated interband interactions. *Nat. Commun.* **5**, 3493 (2014).

2. Mahan, G. D. *Many-Particle Physics* (Springer Science & Business Media, 2000).

3. Zhang, W. *et al.* Ultrafast quenching of electron-boson interaction and superconducting gap in a cuprate. *Nat. Commun.* **5**, 4959 (2014).

4. Ishida, Y. *et al.* Quasi-particles ultrafastly releasing kink bosons to form Fermi arcs in a cuprate superconductor. *Sci. Rep.* **6**, 18747 (2016).

5. Chamon, C. Solitons in carbon nanotubes. *Phys. Rev. B* **62**, 2806–2812 (2000).

6. Saito, R., Dresselhaus, G. & Dresselhaus, M. S. *Physical Properties of Carbon Nanotubes.* (Imperial College Press, 1998).

7. Mohr, M. *et al.* Phonon dispersion of graphite by inelastic x-ray scattering. *Phys. Rev. B* **76**, 035439 (2007).

8. Pietronero, L., Strässler, S., Zeller, H. R. & Rice, M. J. Electrical conductivity of a graphite layer. *Phys. Rev. B* **22**, 904–910 (1980).
